# Derivation and validation of a composite scoring system (SAVED_2_) for prediction of unfavorable modified Rankin scale score following intracerebral hemorrhage

**DOI:** 10.3389/fneur.2023.1112723

**Published:** 2023-02-22

**Authors:** Craig I. Coleman, Mauricio Concha, Bruce Koch, Belinda Lovelace, Mary J. Christoph, Alexander T. Cohen

**Affiliations:** ^1^Department of Pharmacy Practice, University of Connecticut School of Pharmacy, Storrs, CT, United States; ^2^Evidence-Based Practice Center, Hartford Hospital, Hartford, CT, United States; ^3^Department of Neurology, Sarasota Memorial Hospital, Sarasota, FL, United States; ^4^Alexion, AstraZeneca Rare Disease, Boston, MA, United States; ^5^Guy's and St. Thomas' Hospitals, King's College London, London, United Kingdom

**Keywords:** cerebral hemorrhage, risk prediction, modified Rankin scale score, SAVED_2_ score, hemorrhagic stroke

## Abstract

**Objective:**

To develop a composite score for predicting functional outcome post–intracerebral hemorrhage (ICeH) using proxy measures that can be assessed retrospectively.

**Methods:**

Data from the observational ERICH study were used to derive a composite score (SAVED_2_) to predict an unfavorable 90-day modified Rankin scale (mRS) score. Independent predictors of unfavorable mRS were identified *via* multivariable logistic regression and assigned score weights based on effect size. Area under the curve (AUC) was used to measure the score's discriminative ability. External validation was performed in the randomized ATACH-2 trial.

**Results:**

There were 2,449 patients from ERICH with valid mRS data who survived to hospital discharge. Predictors associated with unfavorable 90-day mRS score and their corresponding point values were: age ≥70 years (odds ratio [OR], 3.8; 1-point); prior stroke (OR, 2.8; 1-point); need for ventilation (OR, 2.7; 1-point); extended hospital stay (OR, 2.7; 1-point); and non-home discharge location (OR, 5.3; 2-points). Incidence of unfavorable 90-day mRS increased with higher SAVED_2_ scores (*P* < 0.001); AUC in ERICH was 0.82 (95% CI, 0.80–0.84). External validation in ATACH-2 (*n* = 904) found an AUC of 0.74 (95% CI, 0.70–0.77).

**Conclusions:**

Using data collected at hospital discharge, the SAVED_2_ score predicted unfavorable mRS in patients with ICeH.

## Introduction

Functional outcome following stroke is clinically meaningful and of major relevance to patients. Tools, such as the modified Rankin scale (mRS), have been developed for the accurate assessment of post-stroke functional outcome (1–3). However, functional outcome data are often unavailable or difficult to collect from real-world sources, such as electronic health records and administrative claims databases.

When long-term functional outcome data are not available, surrogate or proxy measures provide alternative methods to assess post-stroke functional outcome. Proxy measures have included discharge destination (4) and home-time calculations (5), both of which strongly correlate with functional outcome measures between 3 and 12 months post-stroke (6). For instance, in a systematic literature review, Costa et al. found that being discharged to a location other than home was associated with an unfavorable mRS score among the 2 studies that assessed this relationship and that increased home-time post-stroke was associated with improved functional outcomes (6). The majority of proxy measure evaluations have been performed in ischemic stroke populations, while proxy measures in hemorrhagic stroke have not been well characterized.

Composite risk models/scores are used to combine various known risk factors and translate them into a more easily interpretable risk assessment of an individual experiencing a particular outcome (7). Here, we sought to develop a composite scoring system to predict post-intracerebral hemorrhage (ICeH) functional outcome using various proxy measures and risk factors that can be assessed retrospectively with ease and accuracy. By developing a score to predict functional outcomes, we hope to support researchers in better characterizing outcomes post-ICeH among large populations where long-term measures of functional status may not be available.

## Materials and methods

### Study population

We analyzed individual patient-level data from 2 distinct, prospective clinical studies funded by the National Institute of Neurological Disorders and Stroke (Table 1).

**Table 1:** Summary of the ERICH and ATACH-2 study designs and overall patient populations.

	**ERICH (8, 10)**	**ATACH-2 (12)**
**Study design**	Multicenter, prospective, case-control study	Randomized, multicenter, open-label trial
**Key inclusion criteria**	• Adults with spontaneous ICeH, including critically ill patients • Anticoagulation prior to ICeH was permitted	• Adults with spontaneous supratentorial ICeH • Volume < 60 mL • GCS score ≥5 • INR < 5
**Key exclusion criteria**	• Malignancies that lead to coagulopathy • Dural venous sinus thrombosis-associated hemorrhage • Hemorrhages attributable to vascular malformations, aneurysms, tumors, or hemorrhagic conversion of recent ischemic stroke	• ICeH related to trauma • ICeH located in infratentorial regions, such as the pons or cerebellum • IVH associated with intraparenchymal hemorrhage and blood completely fills 1 lateral ventricle or more than half of both ventricles • Use of oral anticoagulants within the past 48 h • Pre-morbid disability requiring assistance in ambulation or activities of daily living
**Baseline characteristics**	***N*** **=** **2,568**	***N*** **=** **1,000**
Mean age, years	62.4	61.9
Male, *n* (%)	1499 (58.4)	620 (62.0)
**Race**, ***n*** **(%)**		
Asian	0	562 (56.2)
White	860 (33.5)	287 (28.7)
Black	829 (32.3)	131 (13.1)
Hispanic ethnicity, *n* (%)	879 (34.2)	79 (7.9)
**ICeH location**, ***n*** **(%)**		
Deep	1,347 (52.5)	NA
Thalamus	NA	373 (37.8)^*^
Basal ganglia	NA	506 (51.2)^*^
Lobar	800 (31.2)	108 (10.9)^*^
Cerebellar	193 (7.5)	1 (0.1)^*^
Brainstem	134 (5.2)	0
IVH, *n* (%)	1,089 (42.4)	264 (26.7)^*^
**GCS score**		
Mean (SD) GCS score	mRS 0–3: 13.9 (2.5)	NA
	mRS 4–6: 10.8 (4.2)	NA
3–11 GCS score, *n* (%)	NA	147 (14.7)
12–14 GCS score, *n* (%)	NA	294 (29.4)
15 GCS score, *n* (%)	NA	559 (55.9)

^*^N = 988. GCS, Glasgow Coma Scale; ICeH, intracerebral hemorrhage; INR, international normalized range; IVH, intraventricular hemorrhage; mRS, modified Rankin scale; NA, not available; SD, standard deviation.

The Ethnic/Racial Variations of Intracerebral Hemorrhage (ERICH) study was a multicenter, prospective, case-control study designed to recruit 1,000 non-Hispanic White, 1,000 non-Hispanic Black, and 1,000 Hispanic patients with spontaneous ICeH to identify risk factors among different races and ethnicities (8). The ERICH study allowed the inclusion of critically ill patients with ICeH, including those with Glasgow Coma Scale (GCS) score < 5, intraventricular bleeding, and infratentorial bleeds (8–10). ERICH also included patients who had received anticoagulation prior to ICeH, and they comprised 13.9% of the White, 7.2% of the Hispanic, and 4.7% of the Black cohorts (11).

The Antihypertensive Treatment of Acute Cerebral Hemorrhage-2 (ATACH-2) multicenter, randomized, open-label trial evaluated the efficacy of early, intensive, antihypertensive intravenous nicardipine treatment in 1,000 patients with spontaneous ICeH (12). The study excluded patients with hemorrhage ≥60 mL, GCS score < 5 at emergency department arrival, infratentorial bleeding (e.g., pons or cerebellum) and intraventricular extension, international normalized range (INR) >1.5, use of dabigatran or other oral anticoagulants, or pre-morbid disability requiring assistance in ambulation or activities of daily living (ADL). The primary outcome was death or disability (mRS score of 4–6, on a scale ranging from 0 [no symptoms] to 6 [death]) at 3 months after randomization. Details of the study design, patient populations, and methods, including those used to determine ICeH location and volume, have been previously described for both the ERICH (8) and ATACH-2 (12) studies.

**Figure 1:**
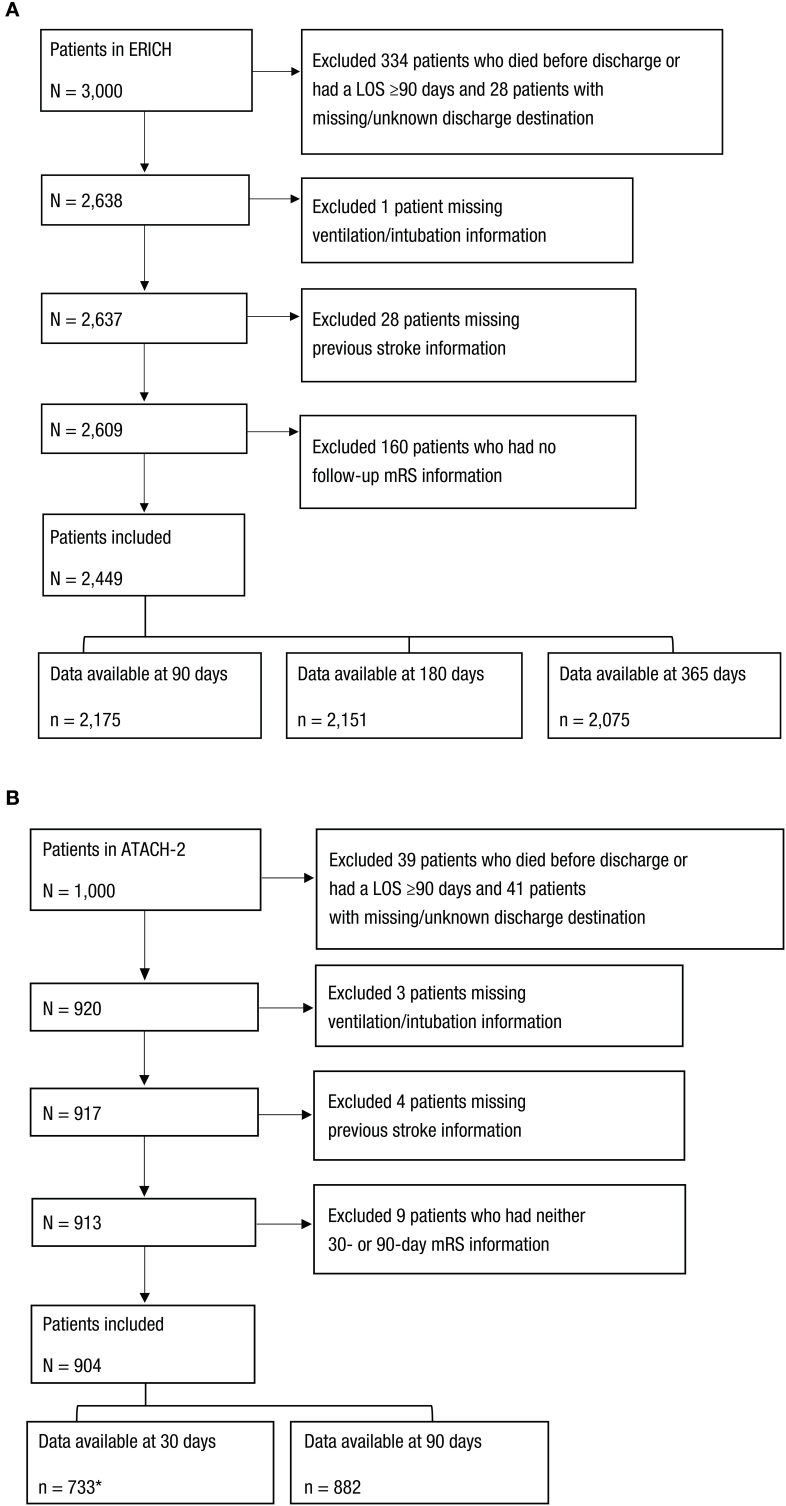
Selection of Patients From the **(A)** ERICH and **(B)** ATACH-2 Studies. *Excluded patients with a LOS ≥30 days. LOS, length of stay; mRS, modified Rankin scale.

Both ERICH (8) and ATACH-2 (12) reported mRS score at time points ranging from 1 month to ≥6 months post–hemorrhagic stroke. All patients in ERICH and ATACH-2 were eligible for inclusion in the current study if they survived to discharge and had a total hospital length of stay (LOS) < 90 days. Patients were excluded if discharge destination data, age, mRS score, need for intubation/ventilation, hospital LOS, or prior stroke history were missing or recorded as “unknown.” Attrition diagrams for ERICH and ATACH-2 are shown in Figure 1.

### Outcomes

Based on proxy measures identified in a prior systematic literature review (6) and available ERICH and ATACH-2 data, potential proxy measures of functional status assessed in this study included discharge destination to home (including home healthcare or relative's/friend's home) vs. non-home locations; extended hospital LOS (defined as hospital LOS ≥8 days); and need for endotracheal intubation and ventilation. Functional outcome status was assessed using mRS at 30, 90, and 180 days after ICeH.

### Statistical analysis

The association between proxy measures and unfavorable outcome classification (defined as a mRS score of 3–6) was assessed. The discriminative ability (sensitivity, specificity, positive predictive value [PPV], negative predictive value [NPV], and area under the curve [AUC]) of each proxy measure with unfavorable functional outcome was evaluated.

To derive a composite score to predict 90-day mRS score, we used proxy measures available at hospital discharge (i.e., need for intubation/ventilation, extended LOS, and discharge destination) from the ERICH study. Selected proxy measures were augmented with variables—advanced age (≥70 years) and prior stroke history—as they are included in well-known clinical risk prediction tools for patients with ICeH (i.e., the Intracranial Hemorrhage [ICH] and Functional Outcome in Patients With Primary ICeH [FUNC] scores) (13) and they are also available in real-world datasets.

We used multivariable logistic regression to identify independent predictors of unfavorable mRS score. Based on the effect size, score weights were assigned to significant proxies/covariates to create the prior **S**troke history per chart history, **A**ge ≥70 years, need for **V**entilation, **E**xtended hospital LOS ≥8 days, **D**ischarge to locations other than home (SAVED) score. A simplified score (SAVED_2_) was also derived by assigning points to each covariate based on the relative weight of each predictor's beta-coefficient to make interpretation easier for clinicians at the bedside. Discriminative ability of SAVED_2_ was assessed using AUC. Internal validation of the SAVED_2_ score was performed using data from the ERICH trial and external validation conducted using data from the ATACH-2 trial. All analyses were conducted with IBM SPSS, version 27 (IBM Corp., Armonk, NY).

## Results

### Derivation population (ERICH cohort)

Of the 3,000 patients with ICeH in the ERICH study, 2,449 patients were included in ≥1 of the analyses at 90, 180, or 365 days (Figure 1A). Of the 2,175 patients who survived to discharge and who had valid mRS data at 90 days after ICeH, 2,151 patients had data available at 180 days and 2,075 patients had data available at 365 days.

Nearly one-third of the patients were aged ≥70 years (30%), the median GCS score was 15 (range, 3–15), and 17% had a prior history of stroke (Table 2). Between 53.4 and 58.1% of patients had an unfavorable mRS score (3–6) at Days 30 through 365. Just over two-thirds of patients (68%) were discharged to a location other than home (Table 2). Using discharge destination as a proxy for unfavorable functional outcome yielded high sensitivity (86%), PPV (range, 67–73%), and NPV (range, 74–76%) at 90, 180, and 365 days (Table 3). More than half of the patients (53%) had an extended hospital LOS (Table 2). Extended LOS was modestly discriminative for predicting unfavorable functional outcome, ranging from 66 to 68% for sensitivity, 60 to 65% for specificity, 66 to 73% for PPV, and 59 to 61% for NPV across the different time points (Table 3). Twenty-eight percent of patients were ventilated/intubated during hospitalization for ICeH (Table 2). Using ventilation/intubation as a proxy for unfavorable functional outcome yielded high specificity (range, 86–87%) and PPV (77–82%), and lower NPV (range, 51–56%) and sensitivity (range, 41–42%) at 90, 180, and 365 days (Table 3).

**Table 2:** Demographics and clinical characteristics of the ERICH and ATACH-2 study populations included in the analysis.

**Characteristic**	**ERICH (derivation cohort) *N* = 2,449**	**ATACH-2 (validation cohort) *N* = 904**
Age ≥70 years, *n* (%)	729 (29.8)	240 (26.5)
Male sex, *n* (%)	1,429 (58.4)	559 (61.8)
White race *n* (%)	1,570 (64.1)	236 (26.1)
**Country**, ***n*** **(%)**		
United States	2,449 (100)	359 (39.7)
Japan	–	285 (31.5)
China	–	127 (14.0)
Other	–	133 (14.8)
Median GCS (range)	15 (3–15)	15 (3–15)
Median intracerebral bleed volume, mL (range)	9.5 (0.0–154.0)	9.7 (0.02–71.0)
Prior history of stroke, *n* (%)	420 (17.1)	142 (15.7)
Need for ventilation/intubation, *n* (%)	691 (28.2)	91 (10.1)
Hospital LOS ≥8 days, *n* (%)	1,306 (53.3)	673 (74.4)
Discharge to location other than home/relative's or friend's home, *n* (%)	1,667 (68.1)	633 (70.0)

GCS, Glasgow Coma Scale; LOS, length of stay.

**Table 3:** Discriminative ability of proxy measures for unfavorable functional outcome (mRS Score 3–6) in ERICH and ATACH-2.

	**Sensitivity, %**	**Specificity, %**	**PPV, %**	**NPV, %**	**Accuracy, %**
**ERICH**					
**Non-home discharge destination**					
90 days	86	56	73	74	74
180 days	86	52	69	74	70
365 days	86	50	67	76	70
**Extended hospital LOS**					
90 days	68	65	73	59	67
180 days	68	62	69	61	65
365 days	66	60	66	61	63
**Ventilation/intubation**					
90 days	41	87	82	51	60
180 days	42	87	80	54	62
365 days	42	86	77	56	62
**ATACH-2**					
**Non-home discharge destination**					
30 days	83	58	80	62	75
90 days	85	48	65	74	68
**Extended hospital LOS**					
30 days	81	37	73	48	66
90 days	81	32	58	59	58
**Ventilation/intubation**					
30 days	14	98	95	37	42
90 days	17	98	92	51	55

LOS, length of stay; mRS, modified Rankin scale; NPV, negative predictive value; PPV, positive predictive value.

### Derivation of SAVED_2_

To make a tool that clinicians can use at the patient bedside, we next derived a composite risk score to predict unfavorable outcome at 90 days for patients with ICeH who survived to discharge. Utilizing the ERICH population as the training database, we identified characteristics independently associated with unfavorable 90-day mRS score (3–6) and assigned points to each covariate based on the relative weight of each predictor's beta-coefficient using multivariable logistic regression. Among the 2,175 ERICH patients with available data, predictors independently associated with unfavorable 90-day mRS scores and their corresponding point values included: prior stroke history (odds ratio [OR], 2.8; 1 point), age ≥70 years (OR, 3.8; 1 point), need for ventilation (OR, 2.7; 1 point), extended hospital LOS ≥8 days (OR, 2.7; 1 point), and discharge to a location other than home (OR, 5.3; 2 points) (Table 4). The use of anticoagulation was also evaluated in the logistic regression model but was not found to be significantly associated with unfavorable mRS score (OR, 1.10; 95% confidence interval [CI], 0.77–1.56), potentially due to low utilization of anticoagulants (< 10%) in the ERICH population.

**Table 4:** Derivation of SAVED_2_ in ERICH: association of covariates with unfavorable mRS score (3–6) upon multivariable logistic regression and corresponding point assignment in SAVED_2_.

	**Beta-coefficient**	**OR**	**Lower 95% CI**	**Upper 95% CI**	**Point assignment**
Stroke history (S)	1.04	2.82	2.09	3.81	1
Age ≥70 years (A)	1.33	3.76	2.95	4.81	1
Ventilation (V)	0.98	2.66	2.03	3.48	1
Extended hospital LOS (E)	1.01	2.75	2.18	3.47	1
Discharge: Not home (D)	1.67	5.32	4.23	6.69	2
Constant	−2.12	–	–	–	–

CI, confidence interval; LOS, length of stay; mRS, modified Rankin scale; OR, odds ratio.

The incidence of unfavorable 90-day mRS scores increased across neighboring and worsening SAVED_2_ scores (*P* < 0.001) (Table 5). Unfavorable mRS scores at 90 days were seen in 36 of 289 patients (12.5%) with a SAVED_2_ score of 0 and in 195 of 207 patients (94.2%) with a SAVED_2_ score of 5 to 6. In the ERICH cohort, SAVED_2_ had an excellent ability to discriminate between patients likely to have an unfavorable (3–6) vs. favorable (0–2) mRS score at 90 days (AUC, 0.82; 95% CI, 0.80–0.84; Table 6). A SAVED_2_ score ≥3 predicted an unfavorable mRS score at 90 days with 81% sensitivity, 71% specificity, 80% PPV, 73% NPV, and 77% overall accuracy.

**Table 5:** Distribution of SAVED_2_ scores in derivation cohort from ERICH.

**SAVED_2_ score**	**N**	**Unfavorable mRS score (3–6) at 90 days, *n* (%)**
0	289	36 (12.5)
1	268	70 (26.1)
2	335	134 (40.0)
3	475	323 (68.0)
4	601	504 (83.9)
5	188	176 (93.6)
6	19	19 (100.0)

Kruskal-Wallis test: *P* < 0.001. mRS, modified Rankin scale.

**Table 6:** Discriminative ability of SAVED_2_ score ≥3 to predict unfavorable outcome (mRS 3–6) in the ERICH (derivation) and ATACH-2 (external validation) cohorts.

	**ERICH**		**ATACH-2**	
	**90 days (derivation)**	**180 days (derivation)**	**30 days (external validation)**	**90 days (external validation)**
Sensitivity, %	81	81	74	78
Specificity, %	71	67	66	58
PPV, %	80	75	82	68
NPV, %	73	73	55	69
Accuracy, %	77	74	72	69
AUC (95% CI)	0.82 (0.80–0.84)	0.80 (0.78–0.82)	0.76 (0.73–0.80)	0.74 (0.70–0.77)

AUC, area under the curve; CI, confidence interval; mRS, modified Rankin scale; NPV, negative predictive value; PPV, positive predictive value.

### External validation population (ATACH-2 cohort)

Of 1,000 patients with ICeH in ATACH-2, 904 patients were included in this analysis (Figure 1B). There were 733 patients with mRS data available at 30 days and 882 patients with mRS data available at 90 days.

About one-quarter of the patients were aged ≥70 years (27%), the median GCS score was 15 (range, 3–15), and 16% had a prior history of stroke (Table 2). Unfavorable mRS scores of 3 to 6 were seen in 67% of patients at Day 30 and 54% of patients at Day 90. Most patients (70%) were discharged to a location other than home (Table 2). When using non-home discharge destination as a proxy for unfavorable mRS, sensitivity (83 and 85%) and PPV (80 and 65%) were high, and specificity (58 and 48%) was lower at 30 and 90 days, respectively (Table 3). Nearly three-quarters of patients (74.4%) had an extended hospital LOS (Table 2). Using extended LOS as a proxy for unfavorable mRS yielded a high sensitivity (81%) and low specificity (37 and 32%) at 30 and 90 days, respectively (Table 3). Ten percent of patients were ventilated/intubated during the study (Table 2). When using ventilation/intubation as a proxy for unfavorable mRS, specificity (98 and 98%) and PPV (95 and 92%) were high, whereas sensitivity was low (14 and 17%) at 30 and 90 days, respectively (Table 3).

### External validation of SAVED_2_

External validation in 882 eligible patients from ATACH-2 suggested that SAVED_2_ maintained good discriminative ability at 90 days (AUC, 0.74; 95% CI, 0.70–0.77; Table 6) within a dataset including patients with different baseline characteristics and bleed severity from the derivation dataset. At shorter (30-day) and longer (180-day) follow-up periods of functional outcome assessment, SAVED_2_ appeared to maintain its discriminative ability as evidenced by AUCs remaining in the clinically useful range, which is defined as ≥0.75 (Table 6).

## Discussion

In this study, we identified proxy measures for assessing functional outcomes post-stroke and developed a novel scoring tool to predict 90-day functional outcomes based on data points that can be captured retrospectively. Our analysis confirmed that selected proxy measures, including discharge destination, extended hospital LOS, and need for mechanical ventilation, previously identified in the acute ischemic stroke literature (4, 14–16) may be useful in predicting functional outcome status in patients following ICeH. Discharge destination was highly sensitive but not as specific, whereas the need for mechanical ventilation had high specificity but lower sensitivity, suggesting that a composite measure including several of these proxy measures could create a stronger overall measure that accounts for the limited sensitivity or specificity of any given measure. We demonstrated that the use of these identified proxy measures as part of a composite risk score (SAVED_2_) had a good-to-excellent ability to discriminate between patients likely to have an unfavorable (3–6) compared to favorable (0–2) mRS score at 90 days.

These findings are important as accurate assessment of post-ICeH functional outcome is essential for both clinical and real-world evidence studies. SAVED_2_ has the potential to serve as a tool to approximate functional outcome post-ICeH when standard outcome measures, such as mRS, are unavailable. The score could also be used to look at how factors, such as the hospital level, treatment patterns, and personal characteristics, relate to functional outcomes. In a clinical setting, SAVED_2_ could be used to predict longer-term, 90-day outcomes at the time of hospital discharge.

While SAVED_2_ appeared to have good discriminative ability at 30 days, there is additional need to confirm whether 1-month mRS score alone can be a dependable and more efficient outcome measure in clinical trials. Additional analysis of ATACH-2 trial data—including evaluation of agreement, weighted kappa, and assessment of utility-weighted mRS at 30- and 90-days after adjustment for potential confounding—could help address this question, as could developing a model to predict 90-day mRS based on 30-day mRS scores and potential covariates included in the SAVED_2_ components.

This study is the first, to our knowledge, to report on the development and validation of a tool to assess functional outcomes among patients with ICeH using variables that can be assessed retrospectively. Unlike many studies that use split validation or just internal validation, we derived the SAVED_2_ score using a large, diverse multiethnic cohort and validated it in a separate cohort, strengthening its generalizability to other external cohorts and populations. However, it is possible that differences in the ERICH and ATACH-2 study populations, such as the broader inclusion criteria used in ERICH to include critically ill patients, may have contributed to the slight discrepancy in AUCs observed between the 2 studies. Another limitation of our study is that the data were derived from and validated in populations with spontaneous ICeH and may not be applicable to patients with ischemic stroke or non-ICeH. Although we excluded patients with missing data on any of the key variables, data completion was high for these fields and few patients had missing data. Another limitation is that we derived SAVED_2_ in the context of patients experiencing spontaneous ICeH in a pre–direct oral anticoagulant (DOAC) era, with fewer than 10% being on oral anticoagulants at the time of the bleed. Also, patients taking anticoagulants were excluded from the ATACH-2 validation cohort. It is unknown how anticoagulant use might impact results, as patients with anticoagulant-related ICeH may experience poorer prognoses compared with patients not on anticoagulants at the time of ICeH (17).

Outcomes for patients with ICeH are very poor. Although it has been decreasing, the mortality burden post-ICeH remains high with a US National Inpatient Sample analysis reporting a 24% inpatient mortality rate (18). Anticoagulant use increases the risk of morbidity and mortality (17). Furthermore, patients who survive the ICeH have a high comorbidity burden. For example, in a German study in 61 patients with DOAC-related ICeH, 28 of 43 survivors (65%) had an unfavorable outcome of mRS 3 to 5, indicating moderate-to-severe disability at 3-month follow-up (19).

Functional outcomes are key for supporting ADL among patients, and we hope that this score can be used to better assess these outcomes among large population-based databases where these data were previously unavailable. To improve outcomes among patients, particularly among those taking anticoagulants, further research is needed to assess the relationship between ICeH and long-term functional outcomes. Utilizing proxy measures such as the SAVED_2_ composite score may enable real-world studies of the long-term functional status associated with ICeH.

## Data availability statement

The original contributions presented in the study are included in the article, further inquiries can be directed to the corresponding author.

## Ethics statement

The studies involving human participants were reviewed and approved by Institutional Review Boards/Ethics Committees at the participating sites. The patients/participants provided their informed consent to participate in those studies.

## Author contributions

Contributed to the design, conceptualization of the study, and interpretation of data: CC, MCo, BK, BL, MCh, and AC. Statistical analysis: CC. All authors participated in drafting and revising the manuscript for intellectual content and approved the final manuscript.
